# Preoperative platelet count predicts posttransplant portal vein complications in orthotopic liver transplantation: a propensity score analysis

**DOI:** 10.1186/s12876-020-01553-z

**Published:** 2021-01-06

**Authors:** Qingshan Li, Yue Wang, Tao Ma, Fenggang Ren, Fan Mu, Rongqian Wu, Yi Lv, Bo Wang

**Affiliations:** 1 National Local Joint Engineering Research Center for Precision Surgery & Regenerative Medicine, Shaanxi Provincial Center for Regenerative Medicine and Surgical Engineering, Institute of Advanced Surgical Technology and Engineering, The First Affiliated Hospital of Xi’an Jiaotong University, No. 277, West Yanta Road, Xi’an, 710061 Shaanxi Province China; 2 Department of Hepatobiliary Surgery, First Affiliated Hospital, Xi’an Jiaotong University, Xi’an, Shaanxi Province China

**Keywords:** Preoperative platelet count, Liver transplantation, Portal vein complication, Inverse probability of treatment weighting

## Abstract

**Background:**

The role of platelets on the prognosis of patients with liver transplantation remains unclear. Thus, we aimed to evaluate the influence of preoperative platelet count on postoperative morbidity after liver transplantation.

**Methods:**

Clinical data of the patients who received liver transplantation from January 2015 to September 2018 were evaluated.

**Results:**

Of the 329 patients included, the average age was 46.71 ± 0.55 years, and 243 were men (75.2%). The incidence of posttransplant portal vein complication was significantly higher in the high platelet count group (> 49.5 × 10^9^/L; *n* = 167) than in the low platelet count group (≤ 49.5 × 10^9^/L, *n* = 162, 12.6% vs. 1.9%). After multivariable regression analysis, high platelet count was independently associated with postoperative portal vein complication (odds ratio [OR]: 8.821, 95% confidence interval [CI]: 2.260 to 34.437). After the inverse probability of treatment weighting analysis, patients in the high platelet count group had significantly higher risk of portal vein complication (OR: 9.210, 95%CI: 1.907 to 44.498, *p* = 0.006) and early allograft dysfunction (OR: 2.087, 95%CI: 1.131 to 3.853, *p* = 0.019).

**Conclusions:**

Preoperative platelet count > 49.5 × 10^9^/L was an independent risk factor for posttransplant portal vein complication and early allograft dysfunction. High preoperative platelet count could be an adverse prognostic predictor for liver transplantation recipients.

## Background

Liver transplantation is considered the only therapeutic option for patients with end-stage liver disease. In the past decades, the outcome of liver transplantation has been dramatically improved with the development of surgical techniques, immunosuppression, and perioperative care [1, 2]. However, posttransplant morbidity incidence remains high, which may affect the survival and quality of life of patients [3]. Thus, better understanding of the contributing factors for posttransplant morbidity is important.

The platelet is a primary factor in various physiological and pathological processes. Recent studies have demonstrated that platelet is involved not only in hemostasis and tissue repair [4], but also in tumor growth and metastasis [5, 6], ischemia/ reperfusion injury [7], and liver regeneration [8, 9]. Patients with severe liver disease for liver transplantation have compromised platelet count and function [10]. These changes may contribute to the physiopathology of liver transplantation. Experimental and clinical studies investigated the role of platelet in candidates for liver transplantation point toward a dualistic result [11–13]. Although platelet is indispensable for liver tissue repair after liver transplantation, platelet can also contribute to graft injury through ischemia/ reperfusion injury. However, most of previous studies focused on platelet posttransplant, and the effect of preoperative platelet count on liver transplantation recipients has not been fully investigated. Therefore, this retrospective study aimed to determine the relationship between preoperative platelet count and outcome after adult liver transplantation.

## Methods

### Patients and data sources

We retrospectively assessed all adult patients (age ≥ 18 years old) who received donation after cardiac death and underwent orthotopic liver transplantation (OLT) from January 2015 to September 2018 at the First Affiliated Hospital, Xi’an Jiaotong University. This paper complies with the STROBE reporting guideline for observational studies.

Clinical data of these patients, including demographic features, donor information, perioperative laboratory values, intraoperative variables, and postoperative complications, were obtained from the electronic medical records. A total of 346 consecutive patients had undergone OLT in our hospital, of which 329 patients were included in this analysis. Patients with missing platelet count data (*n* = 3), re-transplantation (*n* = 8), and incomplete postoperative laboratory values (*n* = 6) were excluded from this study. This retrospective study was approved by our institutional review board (XJTU1AF2015LSL-057), and the requirement for informed consent was waived.

### Outcome parameters

The median follow-up was 16.8 (interquartile range: 9.2–28.8) months. The primary outcome measure was portal vein complication, including portal vein thrombosis and portal vein stenosis, after OLT. Portal vein complication was diagnosed with ultrasonography and computed tomographic scan. The secondary outcomes were overall survival, hepatic artery thrombosis, biliary strictures, early allograft dysfunction (EAD), in-hospital mortality, prolonged intensive care unit (ICU) stay, and postoperative hospital stay.

EAD was defined as the presence of at least one of the following laboratory parameters 7 days after OLT: bilirubin ≥10 mg/dL on day 7, international normalized ratio ≥ 1.6 on day 7, and alanine or aspartate aminotransferases > 2000 IU/L within the first 7 days [14]. Prolonged ICU stay was defined as postoperative stay in the ICU for more than 7 days [15].

### Statistical analysis

Categorical variables were reported as numbers and percentages and compared by the chi-squared analysis or Fisher’s exact test as appropriate. Continuous data were tested by the Kolmogorov-Smirnov test for normality. Normally and abnormally distributed variables were expressed as mean ± standard deviation (SD) and median (interquartile range, IQR) and were compared by Student’s *t*-test and Mann-Whitney rank-sum test, respectively. The optimal cut-off value of preoperative platelet count was calculated by receiver operating characteristic (ROC) curve analysis by using the Youden index according to the incidence of portal vein complication after OLT. Kaplan-Meier estimation and log-rank test were used to analyze the overall survival between different groups. Univariate and multivariate analyses of prognostic factors were performed using logistic regression analysis.

The inverse probability of treatment weighting (IPTW) method was used in this study to reduce the bias in patient selection. We conducted a logistic regression model to estimate propensity scores. The covariates in the model included donor age, recipient body mass index, red blood cell count, leukocyte count, lymphocyte count, total bilirubin, albumin, prothrombin time (PT), activated partial thromboplastin time (APTT), model for end-stage liver disease (MELD) score, coexisting conditions, operation time, anhepatic phase, intraoperative blood loss, total input quantity, and cold ischemia time. Propensity scores were estimated for each patient, and stabilized IPTW weights were created [16]. The detailed method of IPTW was described in our previous study [15]. The power of our sample was 97% at an alpha of 0.05, sample size of 167 and 162 in the high and low preoperative platelet count group respectively, and an odds ratio in the group proportions of 7.6. Two-sided *P*-values < 0.05 were considered statistically significant. All statistical analyses were performed using SPSS Statistics 22.0 software (IBM Corporation, Armonk, NY, United States).

## Results

### Patient demographics and cut-off value of preoperative platelet count

Of the 329 patients included in this analysis, 248 were men (75.4%), and 81 were women (24.6%). The mean age was 46.71 ± 0.55 years. Pretransplant diagnosis included viral hepatitis (*n* = 170, 51.7%), hepatocellular carcinoma (*n* = 95, 28.9%), alcoholic cirrhosis (*n* = 8, 2.4%), primary biliary cirrhosis or autoimmune liver disease (*n* = 20, 6.0%), and other reasons (*n* = 36, 11.0%). Thirty-two patients died during the follow-up period. The median preoperative platelet count was 50 × 10^9^/L (IQR, 34 × 10^9^/L to 86 × 10^9^/L).

**Fig. 1:**
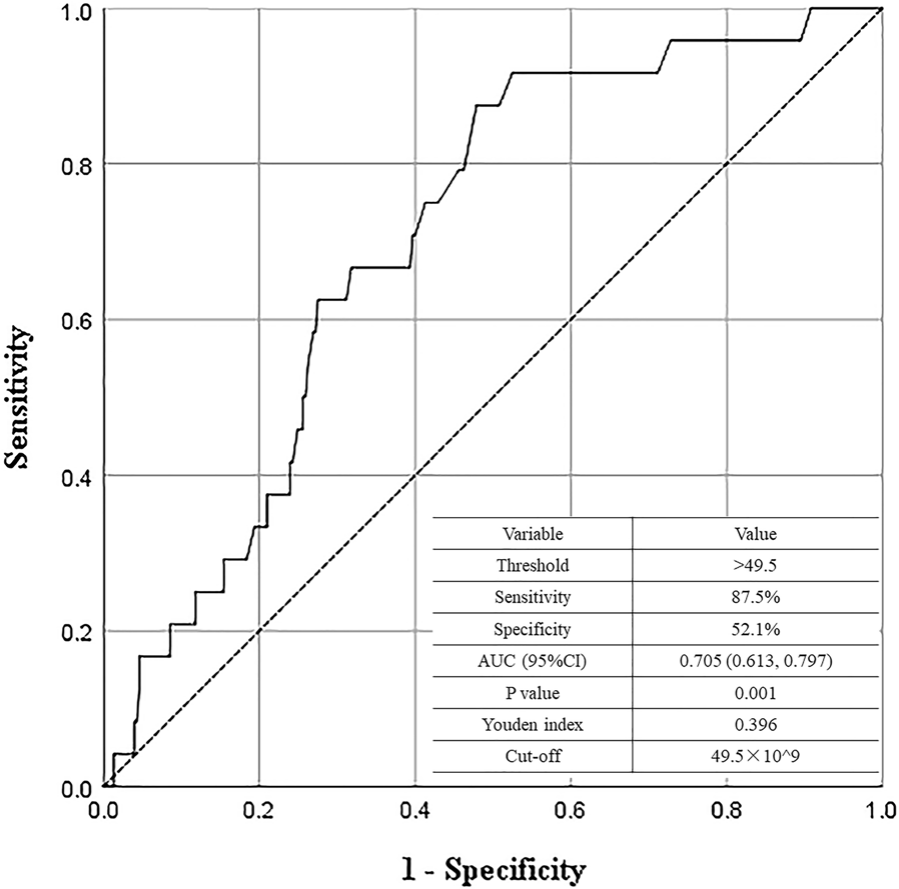
Determination of optimal preoperative platelet count cut-off value by receiver operating characteristic analysis

The diagnostic ability of preoperative platelet count for post-OLT portal vein complication was determined by ROC curve analysis. Based on the data, preoperative platelet count showed a good prediction ability for post-OLT portal vein complication (AUC = 0.705, 95%CI 0.613 to 0.797, *p* = 0.001). The optimal cut-off value for preoperative platelet count was 49.5 × 10^9^/L with the maximum Youden index of 0.396 (sensitivity = 87.5%, specificity = 52.1%, Fig. 1). According to the cut-off value, 167 patients (50.8%) were assigned to the low platelet count group (> 49.5 × 10^9^/L) and 162 patients (49.2%) were assigned to the high platelet count group (≤ 49.5 × 10^9^/L). The distribution of preoperative platelet count for each patient in different groups was shown in Figure S1.

### Baseline characteristics

The baseline variables of the two groups are shown in Table 1. The donor age was a little younger in the high platelet count group (45.22 ± 1.16 years old) than in the low platelet count group (46.12 ± 1.12 years old). Patients in the high platelet count group had a higher blood cell counts (*p* < 0.001) and higher rate of hypertension (12% vs 9.3%, *p* = 0.002) and preoperative splenectomy (12% vs. 4.9%, *p* = 0.022). However, PT, APTT, and MELD scores were significantly higher in the low platelet count group (*p* < 0.05). In terms of intraoperative variables, patients in the low platelet count group experienced a longer operation time (*p* = 0.014), a longer anhepatic phase (*p* = 0.006), and more blood loss and total input amount (*p* < 0.001). Other parameters analyzed had no significant differences between the two groups (Table 1).

**Table 1:** Characteristics of demographic and clinical features of the patients

Variables	PLT > 49.5 × 10^9/L (*n* = 167)	PLT ≤ 49.5 × 10^9/L (*n* = 162)	*P* value
Donor features			
Donor age (years)	45.22 ± 1.16	46.12 ± 1.12	0.009
Donor gender (male/female)	122/45	126/36	0.32
Donor BMI (kg/m2)	22.08 (20.07, 24.24)	22.04 (19.59, 24.18)	0.469
Demographic features			
Age (years)	47.98 ± 0.74	45.18 ± 0.81	0.609
Gender (male/female)	142/25	141/21	0.60
BMI (kg/m2)	22.05 (20.28, 24.22)	22.86 (20.70, 24.88)	0.053
Clinical features			
Preoperative laboratory values			
Creatinine (μmol/L)	60.00 (45.50, 68.40)	55.00 (46.00, 69.00)	0.556
BUN (mmol/L)	4.54 (3.52, 6.61)	4.66 (3.72, 6.10)	0.525
Red cell (× 10^12^/L)	3.44 (3.05, 4.17)	3.08 (2.73, 3.83)	< 0.001
Leukocyte (×10^9^/L)	4.63 (3.35, 6.54)	3.01 (2.11, 4.41)	< 0.001
Lymphocyte (×10^9^/L)	0.80 (0.48, 1.35)	0.47 (0.30, 0.70)	< 0.001
ALT (U/L)	33.83 (23.00, 65.00)	31.50 (23.00, 48.75)	0.204
AST (U/L)	44.00 (31.00, 82.00)	42.00 (30.00, 62.75)	0.103
Total bilirubin (μmol/L)	35.60 (20.20, 83.90)	54.35 (30.00, 112.15)	0.002
Albumin (g/L)	36.90 (32.71, 42.75)	34.35 (31.20, 39.00)	0.005
PT (s)	17.20 (15.05, 19.55)	19.15 (17.50, 21.88)	< 0.001
APTT (s)	43.00 (39.00, 47.90)	48.05 (42.80, 52.60)	< 0.001
Hepatic features			
MELD	11.00 (7.00, 17.00)	13.50 (9.00, 19.00)	0.043
Etiology			0.031
Viral hepatitis (%)	73 (43.7%)	97 (59.9%)	
Alcoholic cirrhosis (%)	3 (1.8%)	5 (3.1%)	
Hepatocellular carcinoma (%)	59 (35.3%)	36 (22.2%)	
Primary biliary cirrhosis & Autoimmune liver disease (%)	12 (7.2%)	8 (9.8%)	
Other (%)	20 (12.0%)	16 (9.9%)	
Coexisting conditions			
Smoking (%)	56 (33.5%)	50 (30.9%)	0.605
Drinking (%)	25 (15.0%)	36 (22.2%)	0.091
Hypertension (%)	20 (12.0%)	5 (3.1%)	0.002
Diabetes (%)	20 (12.0%)	15 (9.3%)	0.424
Pre-operative PVT (%)	32 (19.2%)	25 (15.4%)	0.372
Pre-operative splenectomy (%)	20 (12.0%)	8 (4.9%)	0.022
Intraoperative factors			
Operation time (min)	360.00 (320.00, 420.00)	390.00 (343.75, 421.25)	0.014
Anhepatic phase (min)	47.00 (43.00, 54.00)	50.00 (45.00, 57.25)	0.006
Intraoperative blood loss (mL)	1000 (600, 2000)	1500 (837.50, 2850.00)	< 0.001
Total input amount (mL)	5370 (4500, 6585)	6080 (5235, 8094)	< 0.001
Warm ischemia time (min)	14.00 (10.00, 15.00)	14.00 (10.00, 15.00)	0.733
Cold ischemia time (h)	6.00 (5.00, 6.00)	6.00 (4.00, 6.00)	0.091

### Postoperative outcomes

**Fig. 2:**
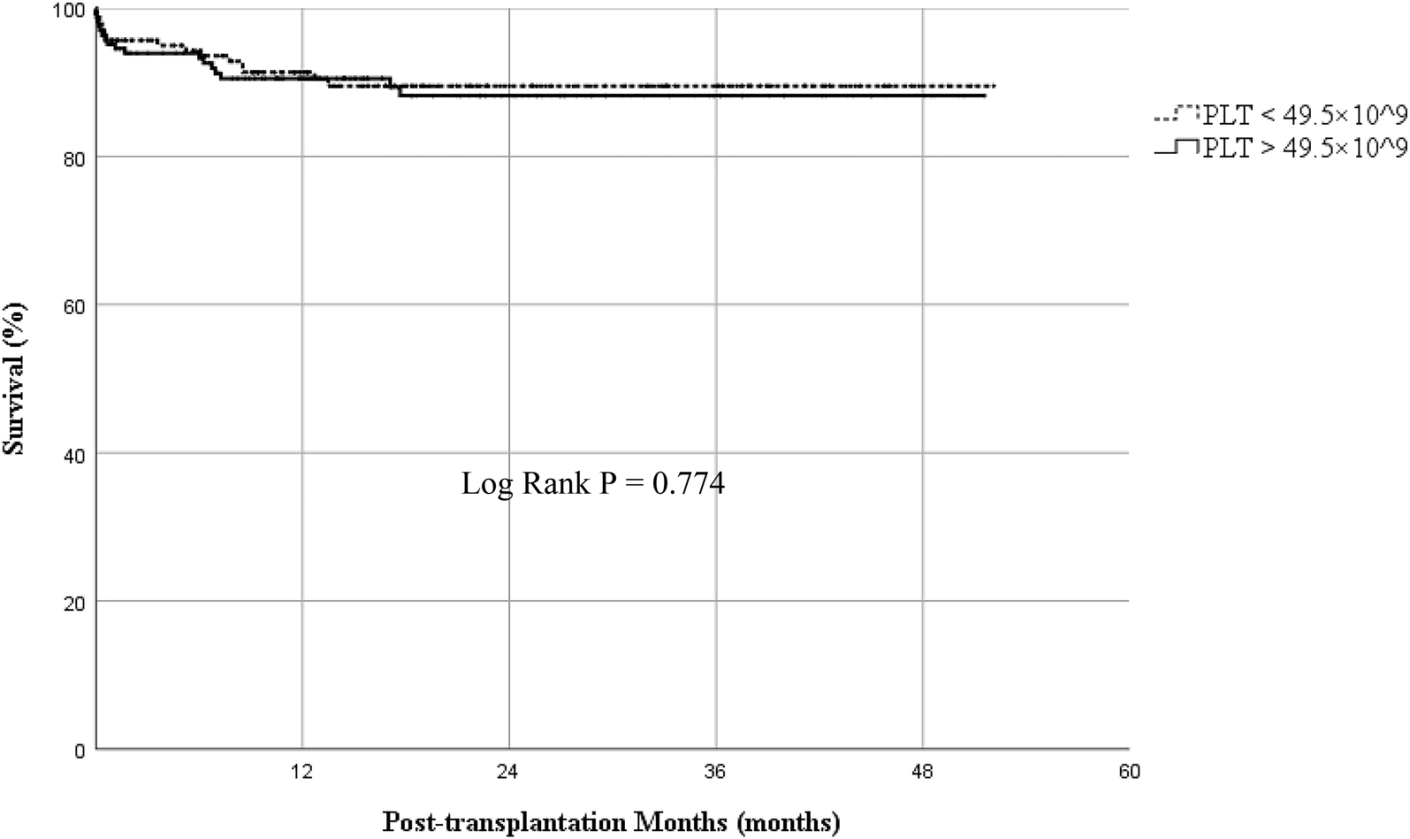
Kaplan-Meier estimation of overall survival according to preoperative platelet count

Postoperative outcomes are shown in Table 2. Portal vein complication occurred in 24 patients after OLT (7.29%). Twenty-one patients (12.6%) in the high platelet count group developed postoperative portal vein complication compared with the three patients (1.9%) in the low platelet count group. The difference was statistically significant (OR 7.623, 95%CI 2.227 to 26.091, *p* < 0.001). Kaplan-Meier analysis showed comparable overall survival rate between the two groups (log rank *p* = 0.774, Fig. 2). Similarly, the incidence of hepatic artery thrombosis, biliary stricture, EAD, in-hospital mortality, and prolonged ICU stay did not show significant difference between the two groups. In addition, multivariable regression was used to adjust the imbalance in potential confounding variables. In the multivariable analysis, high preoperative platelet count was independently associated with a higher incidence of postoperative portal vein complication (OR 8.821, 95% CI 2.260 to 34.437, *p* = 0.002, Table 2).

**Table 2:** Primary and secondary outcomes in two groups

Outcomes	PLT > 49.5 × 10^9/L (*n* = 167)	PLT ≤ 49.5 × 10^9/L (*n* = 162)	Univariate analysis		Multivariable analysis	
			*p*	OR (95% CI)	*p*	OR (95% CI)
Portal vein complications (%)	21 (12.6%)	3 (1.9%)	< 0.001	7.623 (2.227, 26.091)	0.002	8.821 (2.260, 34.437)
Hepatic artery thrombosis (%)	5 (3.0%)	4 (2.5%)	0.770	1.219 (0.321, 4.623)		
Biliary strictures (%)	22 (13.2%)	22 (13.6%)	0.914	0.966 (0.512, 1.822)		
Early allograft dysfunction (%)	36 (22.0%)	26 (16.4%)	0.203	1.439 (0.822, 2.518)		
In-hospital mortality (%)	9 (5.4%)	6 (3.7%)	0.464	1.481 (0.515, 4.260)		
Prolonged ICU stay (%)	74 (44.3%)	63 (38.9%)	0.319	1.250 (0.806, 1.940)		
Post-operative hospital stay (days)	17 (14, 22)	18 (14, 24)	0.445	/		

*Abbreviations*: *ICU* intensive care unit

### Baseline characteristics and postoperative outcomes after IPTW analysis

After IPTW adjustment, the baseline variables between the high platelet count group and low platelet group were comparable (Table 3). As regards postoperative outcomes, the patients in the high platelet count group had higher incidence of portal vein complication (OR 8.296, 95%CI 1.855 to 37.096, *p* = 0.001) and EAD (OR 1.925, 95% CI 1.098 to 3.374, *p* = 0.022) than those in the low platelet count group. After adjustment by the multivariable regression logistic analysis, the risk of portal vein complication (OR 9.210, 95% CI 1.907 to 44.498, *p* = 0.006) and EAD (OR 2.087, 95% CI 1.131 to 3.853, *p* = 0.019) was also higher in the high platelet count group (Table 4).

**Table 3:** Characteristics of demographic and clinical features of the patients after IPTW

Variables	PLT > 49.5 × 10^9/L (*n* = 167)	PLT ≤ 49.5 × 10^9/L (*n* = 162)	*P* value
Donor features			
Donor age (years)	47.04 ± 1.15	46.58 ± 1.26	0.772
Donor gender (male, %)	152 (86.4%)	120 (86.3%)	0.993
Donor BMI (kg/m2)	22.49 (20.52, 24.22)	22.86 (20.64, 24.69)	
Demographic features			
Age (years)	47.39 ± 0.70	45.30 ± 0.87	
Gender (male, %)	136 (77.3%)	109 (78.4%)	0.808
BMI (kg/m2)	22.80 (20.20, 24.22)	22.05 (19.68, 24.32)	0.542
Clinical features			
Preoperative laboratory values			
Creatinine (μmol/L)	59.72 (44.74, 72.00)	56.00 (47.00, 66.37)	0.552
BUN (mmol/L)	4.40 (3.38, 6.93)	4.77 (3.88, 6.10)	0.954
Red cell (×10^12^/L)	3.57 (3.04, 4.01)	3.29 (2.82, 3.97)	0.012
Leukocyte (×10^9^/L)	3.57 (2.77, 5.43)	3.27 (2.35, 5.37)	0.119
Lymphocyte (×10^9^/L)	0.58 (0.45, 1.03)	0.53 (0.34, 0.89)	0.351
ALT (U/L)	35.11 (21.00, 70.00)	30.37 (23.96, 48.59)	0.669
AST (U/L)	44.00 (31.00, 85.00)	42.66 (29.00, 63.00)	0.915
Total bilirubin (μmol/L)	41.73 (23.85, 110.03)	55.20 (28.76, 114.20)	0.333
Albumin (g/L)	36.82 (32.71, 41.80)	35.75 (31.80, 41.14)	0.190
PT (s)	18.14 (15.51, 21.42)	18.50 (16.90, 21.80)	0.389
APTT (s)	45.00 (39.50, 50.20)	47.25 (41.76, 52.22)	0.904
Hepatic features			
MELD	12.00 (6.74, 19.15)	13.00 (9.00, 17.00)	0.713
Etiology			0.298
Viral hepatitis (%)	84 (47.7%)	83 (59.7%)	
Alcoholic cirrhosis (%)	6 (3.4%)	3 (2.2%)	
Hepatocellular Carcinoma (%)	53 (30.1%)	33 (23.7%)	
Primary biliary cirrhosis & Autoimmune liver disease (%)	9 (5.1%)	7 (5.0%)	
Other (%)	24 (13.6%)	13 (9.4%)	
Coexisting conditions			
Smoking (%)	64 (36.4%)	39 (28.1%)	0.119
Drinking (%)	45 (25.6%)	29 (20.9%)	0.328
Hypertension (%)	11 (6.3%)	4 (2.9%)	0.259
Diabetes (%)	14 (8.0%)	14 (10.1%)	0.512
Pre-operative PVT (%)	33 (18.6%)	18 (12.9%)	0.172
Pre-operative splenectomy (%)	12 (6.8%)	8 (5.8%)	0.701
Intraoperative factors			
Operation time (min)	389.33 (330.00, 470.00)	386.43 (340.00, 424.11)	0.296
Anhepatic phase (min)	49.00 (44.00, 57.00)	49.00 (45.00, 57.00)	0.285
Intraoperative blood loss (mL)	1500.00 (800.00, 3000.00)	1500.00 (800.00, 2573.16)	0.564
Total input amount (mL)	6072.43 (4785.37, 7820.00)	6000.00 (5120.00, 7913.63)	0.947
Warm ischemia time (min)	12.00 (6.74, 19.15)	13.00 (9.00, 17.00)	0.617
Cold ischemia time (h)	6.00 (5.00, 6.00)	6.00 (4.00, 6.00)	0.476

**Table 4:** Primary and secondary outcomes in two groups after IPTW

Outcomes	PLT > 49.5 × 10^9/L (*n* = 167)	PLT ≤ 49.5 × 10^9/L (*n* = 162)	Univariate analysis		Multivariable analysis	
			*p*	OR (95% CI)	*p*	OR (95% CI)
Portal vein complications (%)	18 (10.2%)	2 (1.4%%)	0.001	8.296 (1.855, 37.096)	0.006	9.210 (1.907, 44.489)
Hepatic artery thrombosis (%)	6 (3.4%)	3 (2.2%)	0.514	1.549 (0.365, 6.577)		
Biliary strictures (%)	16 (9.1%)	18 (12.9%)	0.273	0.668 (0.325, 1.371)		
Early allograft dysfunction (%)	48 (27.1%)	22 (15.8%)	0.022	1.925 (1.098, 3.374)	0.019	2.087 (1.131, 3.853)
In-hospital mortality (%)	9 (5.1%)	4 (2.9%)	0.323	1.722 (0.533, 5.566)		
Prolonged ICU stay (%)	65 (36.9%)	48 (34.5%)	0.659	1.119(0.703, 1.781)		
Post-operative hospital stay (days)	19 (14, 23)	18 (14, 23)	0.964	/		

*Abbreviations*: *ICU* intensive care unit

## Discussion

High morbidity rate remains an unsolved problem that affects the overall survival of patients who underwent liver transplantation. Thus, better understanding of the risk factors associated with posttransplant morbidity is important to improve the prognosis of these patients. Our current data demonstrated the independent association of preoperative platelet count with posttransplant morbidity. In this study, we found that the risk of posttransplant portal vein complication was greater in patients with high preoperative platelet count (> 49.5 × 10^9^/L) after adjustment for confounders in the multivariable regression. Furthermore, a high preoperative platelet count showed an independent association not only with posttransplant portal vein complication but also with EAD after reducing the selection bias with IPTW analysis. To the best of our knowledge, this study is the first to assess the effect of preoperative platelet count on posttransplant outcomes in liver transplant patients by using the IPTW method.

Platelet is critically important in hemostasis and thrombosis. Normally, hemostasis is tightly regulated and stabilized to prevent blood loss in the case of vessel wall damage [17]. In liver transplant recipients with chronic or acute liver disease, various alterations in primary and secondary hemostatic processes may occur [18, 19]. These alterations may disturb the hemostatic balance of prohemostatic and antihemostatic factors and lead to bleeding or thrombotic disorders [20, 21]. In this study, preoperative thrombocytopenia was observed in liver transplantation recipients (median: 50 × 10^9^/L), and among these patients, the risk of postoperative portal vein complication was higher in patients with high preoperative platelet count. This result may be illustrated on the one hand by the alteration of platelet function in these liver transplant recipients. Studies revealed that platelet function in flowing blood is not impaired in cirrhosis patients, and the thrombocytopenia status could be balanced by elevated platelet adhesive protein von Willebrand factor (vWF) level and the inhibition of its regulator [10, 22, 23]. vWF plasma levels can be elevated more than 10-fold in these patients, thereby supporting platelet adhesion under flow conditions [11]. Therefore, patients with high preoperative platelet count on the basis of thrombocytopenia may have a higher risk of thrombosis because of higher platelet number and enhanced platelet function. On the other hand, the incidence of preoperative splenectomy was higher in the high preoperative platelet count group than in the low preoperative count group in our current study (12.0% vs. 4.9%). The number of postoperative platelets in these patients may recover faster, which may affect their coagulation status and increase the risk of thrombosis. Moreover, previous studies showed that the activated platelets after liver transplantation can release highly active microparticles and form pseudopods on their surface that promote their interaction with neutrophils and other immune cells [24]. These interactions may further promote the thromboinflammatory ability of platelets and lead to the incidence of portal vein complications.

In addition to the well-known role in hemostasis, blood platelet has various other nonhemostic functions. There is increasing evidence that platelet plays an important role in tissue regeneration, ischemia/reperfusion injury, inflammation, tumor growth, and angiogenesis [25]. In patients who underwent liver transplantation, all these processes may be involved. Although many studies have investigated the relationship between platelet and prognostic outcomes posttransplant, whether platelet count has beneficial or detrimental effects on these outcomes remains unclear [11]. Lesurtel et al. conducted a retrospective study of 257 consecutive liver transplantation recipients to evaluate the value of platelet count in predicting short- and long-term outcomes after liver transplantation [26]. They found that platelet count < 60 × 10^9^/L on postoperative day 5 (the 60–5 criterion) is an independent factor for severe complications and early graft and patient survival. Similarly, low posttransplant platelet count is an independent predictor of grade IIIb/IV complications, biliary anastomotic stricture, and graft loss after transplantation in retrospective studies [2, 27–30]. On the contrary, positive association between high platelet count and posttransplant morbidity has also been reported. Han et al. found that high preoperative platelet count (> 75 × 10^9^/L) is a better predictor of hepatocellular carcinoma recurrence after living donor liver transplantation compared with inflammation-based scores [31]. This result is in line with our results that high preoperative platelet count can serve as a predictor of poor prognosis after liver transplantation.

Despite our novel results, this study has several limitations. First, this study was conducted in a single transplant center with a small sample size. Therefore, the incidence of some posttransplant complications was too low to obtain a positive result. Second, because of the retrospective nature of this study, the results are subject to selection bias, and we could not eliminate the underlying confounders from unmeasured variables. Although IPTW analysis was used to reduce the selective bias, the results may be affected by unknown factors. Thus, a multi-center prospective study is needed to further illustrate the role of platelets on the prognosis of posttransplant patients. Additionally, we only evaluated the value of preoperative platelet count but not the change in post-transplant platelet count and the function of platelet. In the previous studies, low post-transplant platelet count was generally considered as a risk factor of post-transplant morbidity, which was not assessed in the current study. As such, the relation between preoperative platelet count and post-transplant platelet count needs to be further evaluated to clarified the comprehensive of platelet on the prognosis of patients with liver transplantation.

## Conclusions

In conclusion, we evaluated the relationship between preoperative platelet count and outcome after adult liver transplantation and found that preoperative platelet count > 49.5 × 10^9^/L was an independent risk factor for posttransplant portal vein complication and EAD. These findings suggest that high preoperative platelet count could be an adverse prognostic predictor for liver transplant recipients.

## Supplementary Information

**Additional file 1: Figure S1**. The distribution of preoperative platelet count for each patient. PLT, platelet count.

## Data Availability

All data generated or analyzed during this study are included in this published article and its supplementary information files.

## Abbreviations

OLT: Orthotopic liver transplantation
EAD: Early allograft dysfunction
ICU: Intensive care unit
SD: Standard deviation
ROC: Receiver operating characteristic
IPTW: Inverse probability of treatment weighting
PT: Prothrombin time
APTT: Activated partial thromboplastin time
MELD: Model for end-stage liver disease
vWF: von Willebrand factor
