# Supplementary figures and images for: Preoperative platelet count predicts posttransplant portal vein complications in orthotopic liver transplantation: a propensity score analysis
Source: BMC Gastroenterol. 2021 Jan 6;21:1. doi: 10.1186/s12876-020-01553-z (PMC7789364; doi:10.1186/s12876-020-01553-z)

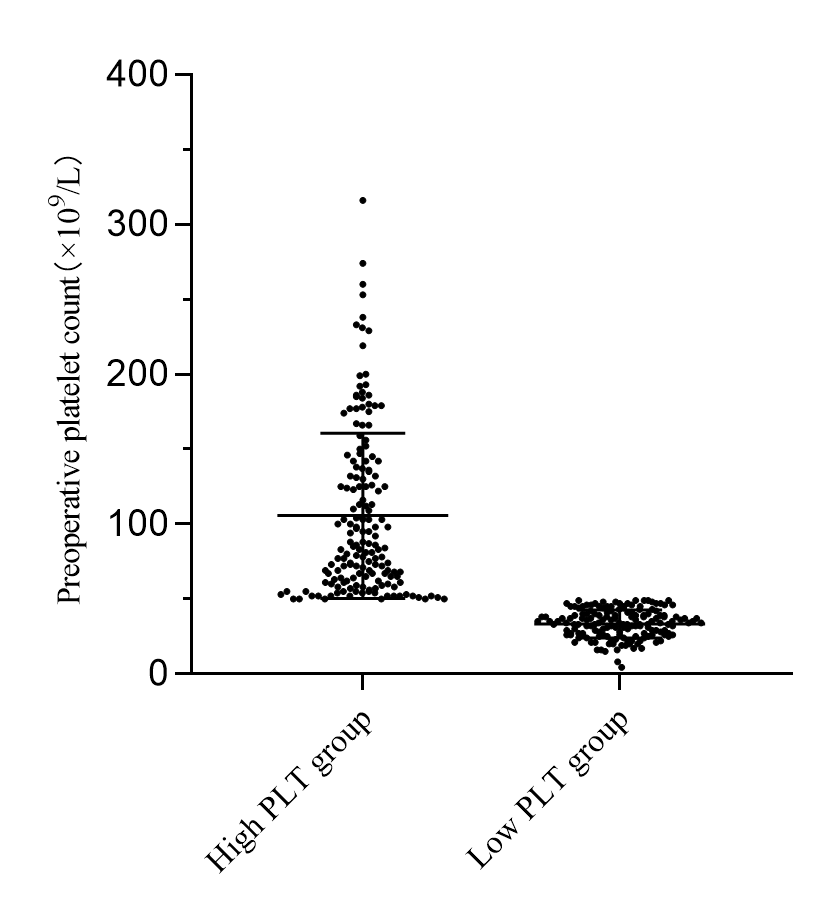

Supplement: Supplementary file 1 — Additional file 1: Figure S1. The distribution of preoperative platelet count for each patient. PLT, platelet count. [file 12876_2020_1553_MOESM1_ESM.tif]
